# Factors related to depression in adults with oral health problems in Spain (2017 to 2020)

**DOI:** 10.3389/fpubh.2024.1364119

**Published:** 2024-02-27

**Authors:** Jesús Cebrino, Silvia Portero de la Cruz

**Affiliations:** ^1^Department of Preventive Medicine and Public Health, University of Seville, Seville, Spain; ^2^Department of Nursing, Pharmacology and Physiotherapy, University of Córdoba, Córdoba, Spain; ^3^Research Group GE10 Clinical and Epidemiological Research in Primary Care, Instituto Maimónides de Investigación Biomédica de Córdoba (IMIBIC), Hospital Universitario Reina Sofía, Córdoba, Spain

**Keywords:** dental health services, depression, oral health, population, health surveys

## Abstract

**Background:**

The need to study the link between gender, depression, and oral health is becoming increasingly evident. This study therefore aimed to determine the prevalence and evolution over time of depression among women and men with oral health problems and to evaluate the association between depression status, lifestyle-related variables health-related variables and use of dental health services in those people.

**Methods:**

We performed a nationwide cross-sectional study on 25,631 adults with oral health problems residing in Spain from the Spanish National Health Survey 2017 and the European Health Survey of Spain 2020, including as the main variable self-reported diagnosis of depression. We analysed independent variables such as lifestyle-related variables, health-related variables, and variables related to dental health services. Sociodemographic characteristics were considered as control variables.

**Results:**

The prevalence of depression among adults with oral health problems in Spain was 7.81% (10.14% for women, 5.39% for men), with a notable decrease from 2017 to 2020 in women. Depressed women had a slightly higher percentage of filled or capped teeth, and had more covers (crowns), bridges or other types of prostheses or dentures, while men had more caries. Women also made more frequent, regular dental visits for check-ups and mouth cleaning, whereas men often needed extractions. Unfavourable associated factors in both genders were: perceiving their health as good, average, poor, or very poor, and having 1–2 and ≥ 3 comorbidities. Conversely, not being a current smoker was related to less likelihood of depression. In women only, not engaging in leisure-time physical activity produced more unfavourable associated factors.

**Conclusion:**

The prevalence of depression among adults with oral health problems in Spain from 2017 to 2020 was 7.81%, but this figure has been steadily decreasing over time. In addition, the favourable and unfavourable associated factors could help us inform health professionals and authorities in order to prevent depression and enhance the care of this population according to gender.

## 1 Introduction

Oral health problems are a global public health concern, with significant health and economic costs (1). Oral health not only affects a person’s quality of life and well-being (2), but also increases the risk of subsequent disorders. Poor oral health has been linked to non-communicable diseases, including cardiovascular diseases and diabetes (3, 4). Additionally, oral health problems have also been associated with mental health conditions, such as depression (5–7).

Globally, depression is currently one of the most prevalent psychiatric disorders, affecting over 300 million people, which is approximately 4.4% of the world’s population (8). In Spain, depression is the most common mental condition, with a lifetime prevalence of 10.5% (9) and twice more prevalent in women compared to males (9.2% vs. 4.0%) (10). Various risk factors contribute to women being more vulnerable than men to the development of mental health problems, such as poor self-esteem, a higher incidence of life stressors, domestic violence and inequalities based on gender (11). Studies have shown that depression disorder is associated with increased healthcare costs (12) and impairments in people’s quality of life (13), along with other concomitant illnesses (14), while it also prevents people from reaching their full potential and impairs human capital (15).

Individuals diagnosed with depression are more likely to develop comorbidities and other systemic illnesses, such as obesity and sleeping difficulties (16). Depression disorder has a substantial impact on oral health through a variety of biological and behavioral factors, including the adoption of dangerous habits such as frequent alcohol consumption, smoking, excessive fat and sugar intake, and sedentary lifestyles (17). Furthermore, past research has shown that depressive illness is connected with less utilization of dental services (16) and increased incidence of periodontitis, tooth loss or dental caries (18, 19). In fact, the highest incidence of dental caries occurs in adults with depression, with an age range from 35 to 44 (20). Thus, xerogenic medications along with a reduced salivary flow and dysregulation of the immune system and salivary immunity associated with depression may increase dental caries and periodontal diseases (21, 22).

To the best of our knowledge, this study is the first to evaluate the relationship between lifestyle-related variables, health-related characteristics, use of dental health services and depression in adults with oral health problems in Spain. The current study aims to address this gap in knowledge. The objectives of this study were therefore to determine the prevalence and evolution over time of depression among women and men with oral health problems and to evaluate the association between depression status, lifestyle-related variables health-related variables and use of dental health services in those people.

## 2 Materials and methods

### 2.1 Design, data source and study population

We conducted a nationwide cross-sectional study using secondary data derived from personalized interviews from the Spanish National Health Survey 2017 (23), conducted between October 2016 to October 2017, and the European Health Survey of Spain 2020 (24), conducted between July 2019 to July 2020. The Ministry of Health, in partnership with the National Institute of Statistics, conducted both surveys among non-institutionalized individuals living mostly in family homes in Spain. To collect the data, the interviews used a stratified three-stage sampling procedure, focusing first on census areas (first stage), then on sections (second stage), and finally on individuals from each household (third stage). Participants were notified by letter, explaining the objective of the survey and its voluntary and anonymous nature, as well as informing them that they would be visited by a qualified interviewer. All participants gave their informed consent. Additional details on the methodology of the SNHS 2017 and EHSS 2020 is available here (25, 26).

**Figure 1:**
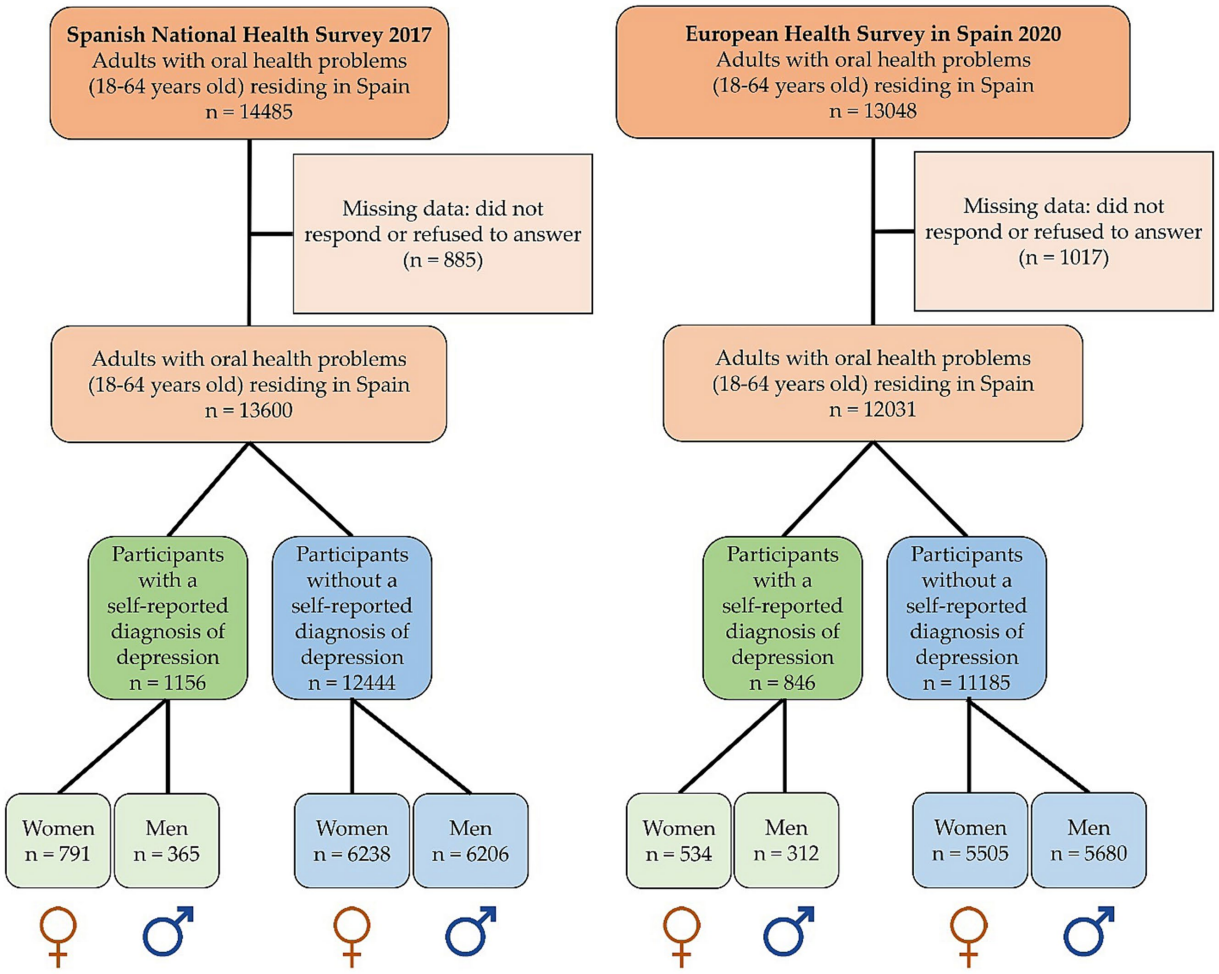
Flowchart of the study population (*N* = 25,631).

In the SNHS and EHSS surveys, if participants were over 18 years old and answered “yes” to the following questions, the presence of oral health problems was confirmed:

In the current study, the participants were those people who were over 18 years old and had, at least, one oral health problem. The presence of oral health problems was confirmed by an affirmative answer to, at least, one of the following questions: (i) “Do you have caries (erosion of the teeth/enamel due to the presence of certain bacteria)?” (ii) “Have you had any teeth extracted?” (iii) “Have you had any teeth filled (fillings),” (iv) “Do your gums bleed spontaneously or when you brush your teeth?” (v) “Do your teeth move?” (vi) “Do you have dentures, caps (crowns), dental splints, or any other kind of prostheses?” (vii) “Do you have any missing teeth that have not been replaced by prostheses?” These items are a valid measure of self-reported oral health problems (27). The initial sample consisted of 27,533 participants with oral health problems (SNHS 2017: 14485 individuals and EHSS 2020: 13048 individuals). However, due to missing data in the selected study variables, 1902 participants were excluded (SNHS 2017: 885 individuals and EHSS 2020: 1017 individuals) during the descriptive and bivariate analyses. Therefore, the final study was based on 25,631 participants: (SNHS 2017: 13600 individuals and EHSS 2020: 12031 individuals). The final sample of SNHS 2017 included 1,156 participants with depression (791 women and 365 men) and 12,444 participants without depression (6,238 women and 6,206 men). Similarly, in the final sample of EHSS 2020, there were 846 participants with depression (534 women and 312 men) and 11,185 participants without depression (5,505 women and 5,680 men) (Figure 1).

### 2.2 Study variables

The dependent variable was the self-reported diagnosis of depression. Thus, participants who answered “yes” when asked: “Have you ever been diagnosed with depression by doctor?” were considered to have self-reported diagnosis of depression.

The independent variables were divided into three categories: (i) lifestyle-related variables, (ii) health-related variables, and (iii) variables related to dental health services:

Lifestyle-related variables: Harmful alcohol consumption (yes, no) according to the latest Spanish Ministry of Health low-risk alcohol consumption guidelines on risks related to alcohol consumption levels, consumption patterns, and the type of beverage (28), current smoking habits (yes, no), and leisure-time physical activity (yes, no).

Health-related variables: Self-perceived state of health (very good, good, average, poor, very poor), body mass index (BMI) (under-weight (BMI < 18.50 kg/m^2^), normal-weight (BMI 18.50–24.99 kg/m^2^), overweight (BMI 25.00–29.99 kg/m^2^)), and obese (BMI ≥ 30 kg/m^2^) (29). Another clinical characteristic was type (s) of chronic disease (s) as assessed by a physician. This variable was classified as the number of comorbidities (none, 1–2, ≥ 3). The chronic diseases included in this study were: hypertension, myocardial infarction, other heart diseases, varicose veins in the legs, osteoarthritis, chronic back pain (cervical), chronic back pain (lumbar), chronic allergy (excluding allergic asthma), asthma (including allergic asthma), chronic bronchitis/emphysema/chronic obstructive pulmonary disease, diabetes, stomach ulcer/duodenum ulcer, urinary incontinence, high cholesterol, cataracts, chronic skin problems, constipation, chronic anxiety, stroke (embolism, cerebral hemorrhage), migraine/frequent headaches, hemorrhoids, malignant tumors, osteoporosis, thyroid problems, prostate problems and menopausal problems.

Variables related to dental health services: Time since last visit to the dentist or dental hygienist (irregular visit: once in the last 12 months or more/never; regular visit: once in the last 3 months or less, 4–6 months ago or 7–11 months ago), check-up (yes, no), mouth cleaning (yes, no), fillings (yes, no), tooth extraction (yes, no), caps, dental splint or other kind of prostheses (yes, no), treatment of gum disease (yes, no), orthodontics (yes, no), fluoride application (yes, no), and implants (yes, no).

Sociodemographic characteristics were considered as control variables. It was included: year of the surveys (2017, 2020); gender (women, men); age groups [divided into three groups, as proposed by Arnett (30): emerging adults aged 18–24 years old, young adults aged 25–44 years old, middle-aged adults aged 45–64 years old]; marital status (single, married, widowed, separated or divorced); nationality (foreign, Spanish); employment status (employed, unemployed); level of education (without studies, primary studies, secondary studies or professional training, university studies); and town of residence (rural, urban). Social class was assigned according to the categories proposed by the Spanish Society of Epidemiology (31). This variable was classified into: Class I (directors and managers of companies with 10 or more employees and professionals normally qualified with university degrees), Class II (directors and managers of companies with less than 10 salaried employees and professionals normally qualified with university degrees other technical support professionals. Athletes and artists). Class III (intermediate professions and self-employed workers), Class IV (supervisors and workers in skilled technical work), Class V (skilled workers in the primary sector and other semi-skilled workers), and Class VI (unskilled workers). As outlined by Fajardo-Bullón et al. (32, 33), we have organized these six original classes were formed into three groups in this study: social classes I and II, social classes III and IV, and social classes V and VI.

### 2.3 Procedure and ethical considerations

The downloaded anonymised data is accessible to the general public via the websites of the National Institute of Statistics and the Ministry of Health (25, 26). According to Spanish law, clearance from the Ethics Committee is not required when using secondary data.

### 2.4 Statistical analysis

The qualitative variables were evaluated using counts and percentages, while the quantitative variables were analysed using arithmetic mean and standard deviation (SD). We used the Kolmogorov–Smirnov test to check the normality of the variables, and Student’s *t* test to compare means. For the contingency tables, we utilised the Chi-square test, with Fisher’s exact test being employed if the number of expected frequencies was over 5. To determine which variables were associated to the presence of depression, we conducted a binary logistic regression, including all the variables in which the univariate test showed a potential association with the dependent variable (*p* ≤ 0.15), with backward selection employed to remove non-significant factors determined by the probability of the Wald statistic. Crude and adjusted Odds Ratios (OR) were calculated with 95% confidence intervals, and the goodness of fit was assessed using the Hosmer–Lemeshow test, with a *p* ≤ 0.05 considered significant. Finally, we carried out the statistical analysis using IBM SPSS Statistical software version 29.0.1.0 (IBM Corp, Armonk, NY, United States), which was licensed to the University of Seville (Spain).

## 3 Results

### 3.1 Characteristics of the study participants

**Figure 2:**
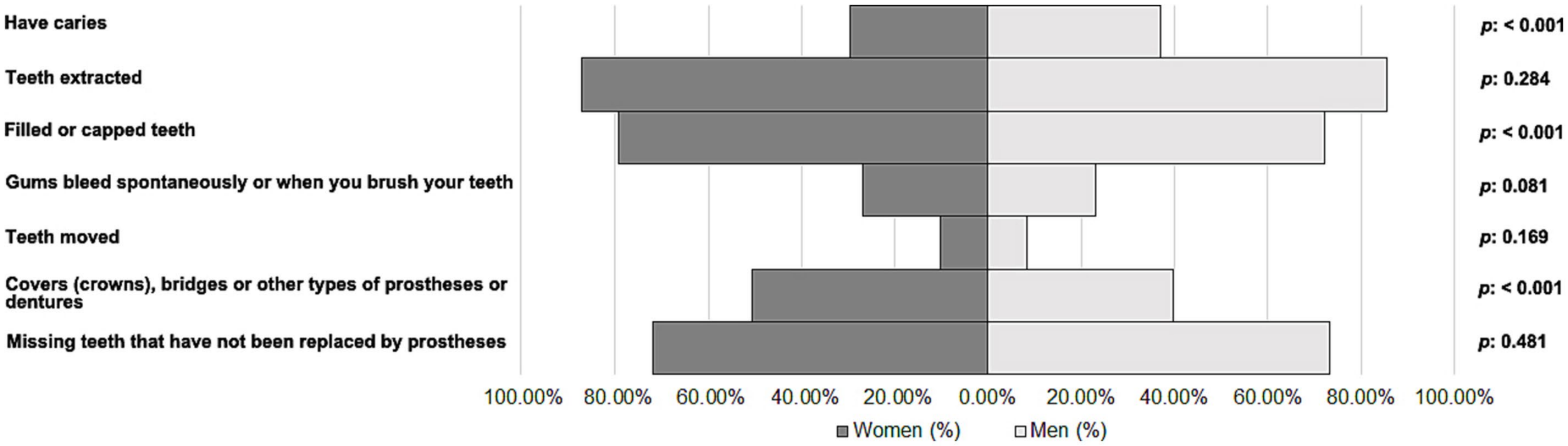
Oral health problems among women and men with depression.

The participants were 25,631 adults with oral health problems residing in Spain. Of them, 7.81% (*n* = 2002) were depressed people (women: *n* = 1,325 [10.14%] and men: *n* = 677 [5.39%]). Figure 2 shows the prevalence of different oral health problems among women and men with depression. Interestingly, women had a slightly lower percentage of caries (29.58%) compared to men (36.93%) (*p* < 0.001). Similarly, women had a slightly higher percentage of filled or capped teeth (79.32%) compared to men (72.08%) (*p* < 0.001). Finally, the results indicated that women had a higher percentage of covers (crowns), bridges or other types of prostheses or dentures (50.64%, compared with 39.73% for men) (*p* < 0.001).

### 3.2 Prevalence and evolution over time of depression in study participants

There was a significant decrease in the prevalence of depression from 2017 (8.50%) to 2020 (7.03%) (*p* < 0.001). This decrease was particularly pronounced in women, decreasing from 11.25% in 2017 to 8.84% in 2020 (*p* < 0.001). However, there was no significant change observed in men, with the prevalence remaining relatively stable from 2017 (5.55%) to 2020 (5.21%) (*p* = 0.389).

### 3.3 Lifestyle-related variables, health-related variables and use of dental services among women and men participants with depression

**Figure 3:**
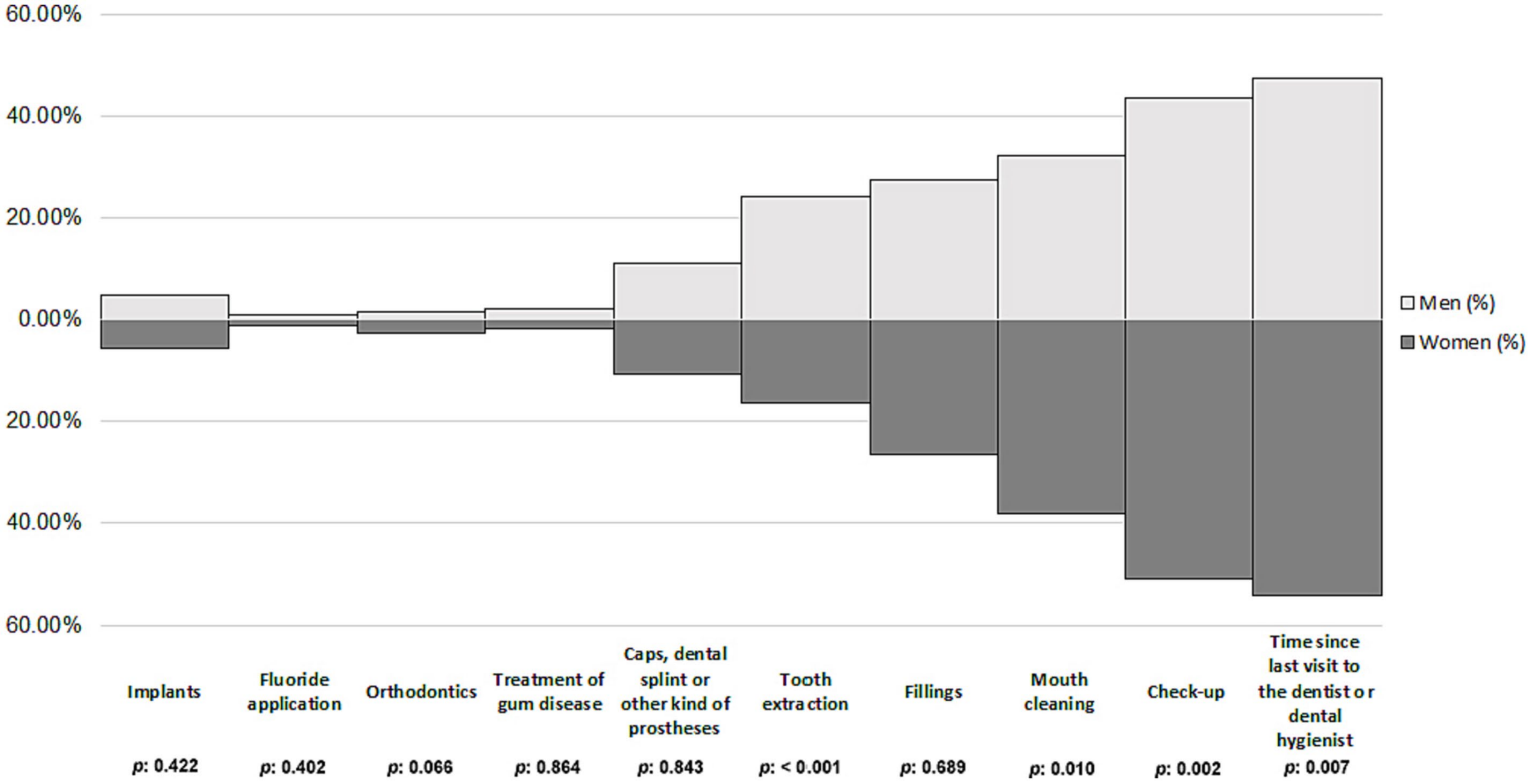
Use of dental health services among women and men participants with depression.

Differences in independent variables were found among women and men participants with depression. Specifically, most of women with depression were not current smokers (*p* < 0.001) and had ≥3 comorbidities (*p* < 0.001), compared to the men. In contrast, men with depression had a higher proportion of overweight individuals (*p* < 0.001) (Table 1). Moreover, t was found that irregular dental visits were more prevalent among men (52.44%) compared to women (46.04%), while regular dental visits were more frequent among women (53.96%) than men (47.56%) (*p* = 0.007). When it came to the use of dental health services, the most common reason was for a check-up (women: 50.72%; men: 43.57%; *p* = 0.002), followed by mouth cleaning (women: 38.19%; men: 32.35%; *p* = 0.010). Additionally, tooth extractions were found to be prevalent, with 16.30% of women and 24.08% of men availing this service (*p* < 0.001) (Figure 3).

**Table 1:** Comparison of women and men with oral health problems and depression as regards sociodemographic characteristics, lifestyle-related variables, and health-related variables (*n* = 2002).

Variables	Total *n* (%) 2002 (100)	Women *n* (%) 1325 (66.18)	Men *n* (%) 677 (33.82)	*p*
Age groups				
18–24 years old	31 (1.55)	16 (1.21)	15 (2.22)	0.222
25–44 years old	502 (25.07)	332 (25.06)	170 (25.11)	
45–64 years old	1,469 (73.38)	977 (73.73)	492 (72.67)	
Marital status				
Single	520 (25.98)	258 (19.47)	262 (38.70)	<0.001
Married	957 (47.80)	673 (50.79)	284 (41.95)	
Widowed	125 (6.24)	114 (8.61)	11 (1.62)	
Separated or divorced	400 (19.98)	280 (21.13)	120 (17.73)	
Nationality				
Foreign	173 (8.64)	138 (10.42)	35 (5.17)	<0.001
Spanish	1829 (91.36)	1,187 (89.58)	642 (94.83)	
Employment status				
Unemployed	1,174 (58.64)	753 (56.83)	421 (62.19)	0.021
Employed	828 (41.36)	572 (43.17)	256 (37.81)	
Level of education				
Without studies	117 (5.84)	77 (5.81)	40 (5.91)	0.674
Primary studies	356 (17.78)	232 (17.51)	124 (18.32)	
Secondary or PT	1,276 (63.74)	840 (63.40)	436 (64.40)	
University studies	253 (12.64)	176 (13.28)	77 (11.37)	
Town of residence				
Rural	414 (20.68)	261 (19.70)	153 (22.60)	0.129
Urban	1,588 (79.32)	1,064 (80.30)	524 (77.40)	
Social class				
Social classes I and II	247 (12.34)	168 (12.68)	79 (11.67)	0.330
Social classes III and IV	597 (29.82)	381 (28.75)	216 (31.90)	
Social classes V and VI	1,158 (57.84)	776 (58.57)	382 (56.43)	
Harmful alcohol consumption				
Yes	65 (3.25)	47 (3.55)	18 (2.66)	0.289
No	1937 (96.75)	1,278 (96.45)	659 (97.34)	
Current smoking habits				
Yes	756 (37.76)	453 (34.19)	303 (44.76)	<0.001
No	1,246 (62.24)	872 (65.81)	374 (55.24)	
Leisure-time physical activity				
Yes	1,091 (54.50)	716 (54.04)	375 (55.39)	0.565
No	911 (45.50)	609 (45.96)	302 (44.61)	
Self-perceived state of health				
Very good	85 (4.25)	55 (4.15)	30 (4.43)	0.542
Good	545 (27.22)	369 (27.85)	176 (26.00)	
Average	833 (41.61)	557 (42.04)	276 (40.77)	
Poor	399 (19.93)	250 (18.87)	149 (22.01)	
Very poor	140 (6.99)	94 (7.09)	46 (6.79)	
Body mass index				
Under-weight	50 (2.50)	39 (2.94)	11 (1.62)	<0.001
Normal-weight	735 (36.71)	512 (38.64)	223 (32.94)	
Overweight	762 (38.06)	465 (35.10)	297 (43.87)	
Obese	455 (22.73)	309 (23.32)	146 (21.57)	
Number of comorbidities				
None	116 (5.79)	60 (4.53)	56 (8.27)	<0.001
1–2	489 (24.43)	285 (21.51)	204 (30.13)	
≥ 3	1,397 (69.78)	980 (73.96)	417 (61.60)	

PT, professional training.

### 3.4 Association between depression status and lifestyle-related variables, and health-related variables in women and men participants

The crude and adjusted ORs for identifying factors related to depression in adults with oral health problems are shown in Tables 2, 3.

**Table 2:** Factors related to depression in women with oral health problems (*n* = 13,068).

Variables	OR (95% CI)	ORa* (95% CI)	*p*
Harmful alcohol consumption			
Yes	Reference		
No	1.57 (1.16–2.13)		
Current smoking habits			
Yes	Reference	Reference	
No	0.69 (0.61–0.78)	0.79 (0.69–0.91)	<0.001
Leisure-time physical activity			
Yes	Reference	Reference	
No	1.59 (1.42–1.79)	1.24 (1.09–1.41)	0.001
Self-perceived state of health			
Very good	Reference	Reference	
Good	2.91 (2.18–3.87)	1.61 (1.19–2.16)	0.002
Average	14.51 (10.93–19.25)	4.35 (3.22–5.88)	<0.001
Poor	33.71 (24.70–46.01)	7.68 (5.49–10.74)	<0.001
Very poor	48.04 (32.59–70.81)	9.10 (6.00–13.81)	<0.001
Body mass index			
Under-weight	Reference		
Normal-weight	0.80 (0.57–1.12)		
Overweight	1.47 (1.05–2.07)		
Obese	2.09 (1.47–2.97)		
Number of comorbidities			
None	Reference	Reference	
1–2	4.72 (3.56–6.25)	3.32 (2.49–4.44)	<0.001
≥3	25.03 (19.20–32.62)	10.08 (7.58–13.40)	<0.001

PT, professional training; OR, odds ratio; *ORa, odds ratio adjusted for all sociodemographic characteristics and health-related variables; 95% CI, 95% confidence interval. Hosmer-Lemeshow test χ^2^ = 14.67, *p* = 0.066; Nagelkerke’s *R*^2^ = 0.305; *p* < 0.001. Model controlled for age groups, marital status, nationality, employment status, level of education, town of residence and social class.

**Table 3:** Factors related to depression in men with oral health problems (*n* = 12,563).

Variables	OR (95% CI)	ORa* (95% CI)	*p*
Harmful alcohol consumption			
Yes	Reference		
No	1.68 (1.04–2.71)		
Current smoking habits			
Yes	Reference	Reference	
No	0.58 (0.50–0.68)	0.64 (0.53–0.76)	<0.001
Leisure-time physical activity			
Yes	Reference		
No	1.73 (1.48–2.02)		
Self-perceived state of health			
Very good	Reference	Reference	
Good	2.62 (1.77–3.86)	1.80 (1.21–2.68)	0.004
Average	16.56 (11.31–24.25)	5.64 (3.75–8.47)	<0.001
Poor	40.20 (26.77–60.38)	10.34 (6.66–16.05)	<0.001
Very poor	57.71 (34.63–96.18)	12.24 (7.05–21.26)	<0.001
Body mass index			
Under-weight	Reference		
Normal-weight	0.30 (0.16–0.58)		
Overweight	0.32 (0.17–0.62)		
Obese	0.42 (0.22–0.81)		
Number of comorbidities			
None	Reference	Reference	
1–2	4.31 (3.20–5.81)	2.92 (2.14–3.99)	<0.001
≥3	19.58 (14.74–25.99)	6.64 (4.81–9.17)	<0.001

PT, professional training; OR, odds ratio; *ORa, odds ratio adjusted for all sociodemographic characteristics and health-related variables; 95% CI, 95% confidence interval. Hosmer-Lemeshow test χ^2^ = 20.23, *p* = 0.010; Nagelkerke’s *R*^2^ = 0.293; *p* < 0.001. Model controlled for age groups, marital status, nationality, employment status, level of education, town of residence and social class.

In both genders, the probability of depression was higher among those who perceived their state of health good, average, poor and very poor (women: OR = 1.61, OR = 4.35, OR = 7.68, OR = 9.10, *p =* 0.002, *p* < 0.001, *p* < 0.001, *p* < 0.001, respectively; men: OR = 1.80, OR = 5.64, OR = 10.34, OR = 12.24, *p* = 0.004, *p* < 0.001, *p* < 0.001, *p* < 0.001, respectively) and who had 1–2 or ≥ 3 number of comorbidities (women: OR = 3.32, OR = 10.08, *p* < 0.001, *p* < 0.001, respectively; men: OR = 2.92, OR = 6.64, *p* < 0.001, *p* < 0.001, respectively). In contrast, not having current smoking habits (women: OR = 0.79, *p* < 0.001; men: OR = 0.64, *p* < 0.001) was associated with a lower probability of depression.

In women exclusively, not doing physical activity during leisure time (OR = 1.24, *p* = 0.001) was associated with a higher probability of depression.

## 4 Discussion

### 4.1 Main findings

The aim of the present study was to investigate the prevalence and evolution of depression over recent years, to examine issues related to oral health and use of dental health services, and to evaluate the link between depression and lifestyle and health-related characteristics in adults with oral health problems in Spain according to gender.

Previous studies reported a prevalence of 9.04% for oral diseases in people with depression in South India (34), and a similar prevalence was observed among American adults (6.93%) (35). These findings are in line with our results, where the prevalence was 7.81%, with a higher percentage observed in women (10.14%) compared to men (5.39%). Similarly, this gender discrepancy agrees with the observations made by Almohaimeed et al. (35), where women showed a higher prevalence of depression than men. Anttila et al. (36) found that depression and poor oral health habits were particularly connected in women, with depressed women having more unfavourable attitudes toward retaining their natural teeth, eating more sweet foods, and foregoing dental procedures more than depressed men. In addition, the prevalence of depression found in the present study decreased from 2017 to 2020, particularly in women. This finding is corroborated by a recent article that revealed a reduction in the burden of depressive symptoms in most European countries, including Spain, between 2006 and 2014 (37). Furthermore, another recent study (38) identified a declining trend in depression among young and middle-aged adults. Additionally, the explanations for these gender differences could be attributed to the fact that women exhibit greater health awareness and are more inclined to seek medical attention than men (39).

Over the years, various studies have found a substantial link between depression and oral health status (40, 41) and use of dental services (42). According to our results, women with depression had a higher percentage of filled or capped teeth and covers (crowns), bridges or other types of prostheses or dentures than depressed men. The subjects might have undergone these treatments for aesthetic reasons, as female patients have been found to be more concerned with the appearance of their teeth (43, 44). In fact, aesthetic considerations play a key role in various functions, such as mastication or speech, which, if lost or impaired, can negatively affect psychological health (45). Meanwhile, caries was more prevalent in men than in women, in contrast to some previous studies (46, 47). This may be because men prefer harder toothbrushes, are less likely to opt for recommended fluoride toothpaste, lack awareness of proper brushing techniques, and often brush more vigorously, potentially leading to gingival damage and recession, and increased risk of caries (48). According to the oral health atlas in Spain (49), brushing at least twice a day with fluoridated toothpaste (preferably after meals), along with daily interdental hygiene using dental floss or interdental brushes, and at least two regular check-ups per year are fundamental practices for maintaining optimal oral health care. In fact, the oral health survey in Spain (50), based on telephone interviews with 1,001 individuals, reveals that 86% of participants brush their teeth at least twice a day, with women brushing more frequently than men. In Spain, only half of the population visits the dentist at least once a year, placing the country at the tail end of European regular dental check-up habits (49). As regards the use of dental health services in the present study, women visited the dentist more regularly, and had more check-ups and had their mouths cleaned more often than men. In contrast, tooth extractions were found to be more prevalent in men than in women. Several studies have confirmed that women tend to make more regular visits to the dentist for routine checkups or planned treatments, while men are more likely to seek dental care for acute problems such as pain (51–53). Stereotyped male attitudes, which have a negative impact on the willingness to seek help (54), along with the perception that the illness is linked to a loss of masculinity (55), could be another possible explanation of why men tend to use dental services less frequently than women.

Based on our results in women exclusively, physical activity is a preventive practice (56), and our study found that women with oral health problems who did not engage in leisure-time physical activity were more likely to develop depression. Other studies support this finding, linking depression, physical activity, and oral health in women (57, 58), and it has also been reported that regular physical activity has a significant impact on women’s management of their mental health, and affects aspects such as health perception, quality of life, and oral health (59), while in men, the link between depression, health and social characteristics seems to be even stronger (60). This would explain why younger males receive less care for mental health issues than elder men and women (61). Moreover, depressed males are more prone to self-medicate (62) and engage in hazardous behaviour (63). This, combined with the fact that men are more inclined to ignore their oral health and have poorer oral hygiene routines (64).

In both genders, a key preventive practice is not smoking, which acted in the present study as a favourable associated factor for depression, in line with another study (35). In contrast, a lower quality of life correlates with poor self-perceived health and is connected to depression (65). According to our results, the likelihood of experiencing depression was higher among people who perceived their health status as good, average, poor, and very poor in both genders. Similarly, a strong connection was found between depression and all other mental illnesses, including the vast majority of somatic diseases (66), which have been found to be an unfavourable associated factor in both genders. People with mental health problems are increasingly prone to experiencing oral health problems, as poor mental health has been linked to other comorbid conditions (67).

### 4.2 Strengths and limitations

Despite the strengths of this study, including a large sample size, randomised population selection, and well-trained collectors of the data, it also has some possible limitations. Firstly, due to the nature of the cross-sectional study, it is not possible to establish causal relationships for the associations observed. Moreover, additional research using longitudinal or experimental studies would be required to establish potential causal relationships. Secondly, although the variable “Have you ever been diagnosed with a particular disease by a doctor?” was self-reported by the participants for various illnesses, including oral health problems and depression, among others, the questions used by SNHS and EHSS were precise. Thirdly, the data collected through interviews can be vulnerable to memory issues or individuals’ tendency to provide socially desirable responses.

### 4.3 Implications for research and practice

The findings of this research, which examines the relationship between sociodemographic characteristics, lifestyle and health-related factors, and variables related to dental health services and depression in adults with oral health problems in Spain, can help inform health professionals and authorities not only about the prevalence and temporal evolution of depression in this population, but also about the favourable and unfavourable factors associated with depression for use in future interventions. For example, given the potential link between the lack of physical activity and issues related to physical health, mental health, and quality of life in women, especially in middle age, our findings suggest the need for measures and strategies to promote physical activity in this group (59), as adequate physical activity can help to alleviate depression in women (68). Meanwhile, not having a partner was linked with higher levels of depression, especially in women (69, 70). Depression symptoms partly overlap with emotional disturbance (71) and may involve feelings of failure, isolation, and sadness (72); however, emotional regulation stands out as a key protective factor in individuals who possess the strategies and skills to be able to use it (73). Therefore, with the information provided in this study, we would emphasise the future need to develop effective interventions in the field of emotional regulation to help reduce the risk of developing depression in the population (74). Finally, concerning Spanish nationality in men as an unfavourable associated factor of depression, the societal demands of the hegemonic male identity, with its focus on hiding one’s feelings and being more reluctant to seek treatment, may make it more difficult to identify depression in males (54, 75). Therefore, incorporating a biopsychosocial approach (76) or the incorporation of feminist approaches to narrative psychotherapies (77) while caring for patients with oral health issues could help to correct the current situation of inequality in clinical care, with the aim of preventing depression (78). It should be noted that the Spanish National Health System (SNS) provides extensive coverage for general health, yet offers minimal oral healthcare for adults, restricting publicly funded clinics to emergency care and oral surgery (dental extractions); the majority of oral health services, involving over 90% of dental professionals, are administered in the private sector (79). Therefore, potential strategies to increase attendance to oral health services could involve the routine incorporation of advice and instructions on oral health in all educational settings (80). In fact, multidisciplinary health professionals should play a role in oral health care as an integral aspect of their patient care for individuals with mental health disorders (81). Other strategies could focus on an awareness campaign highlighting the importance of regular dental visits and check-ups, crucial for maintaining oral health and preventing oral diseases (82).

## 5 Conclusion

The prevalence of depression among adults with oral health problems residing in Spain is currently 7.81% (10.14% for women and 5.39% for men), showing a decrease from 2017 to 2020. Women with depression have a slightly higher prevalence of filled or capped teeth, and have more covers (crowns), bridges, or other types of prostheses or dentures compared to men. Meanwhile, men with depression have more caries than women. As regards the use of dental health services, regular dental visits are more frequent among women than men, and the most common reason for women is for a check-up or mouth cleaning, while for men it is for tooth extractions. Finally, in both genders, adults with oral health problems who perceive their health as good, average, poor, or very poor, and have 1–2 and ≥ 3 comorbidities are more likely to experience depression. In contrast, not being a current smoker is related to a lower likelihood of depression. In women only, not doing leisure-time physical activity is unfavourable associated factors. In order to prevent depression and enhance care in this population, health professionals and authorities should be aware of these associated factors of depression according to gender.

## Data availability statement

Publicly available datasets were analysed in this study. This data can be found here: https://www.sanidad.gob.es/estadisticas/microdatos.do.

## Ethics statement

The requirement of ethical approval was waived by Cordoba Research Ethics Committee. Avda. Menéndez Pidal, s/n 14004 Córdoba (Spain) for the studies involving humans because Cordoba Research Ethics Committee. The studies were conducted in accordance with the local legislation and institutional requirements. The participants provided their written informed consent to participate in this study.

## Author contributions

JC: Conceptualization, Data curation, Formal analysis, Investigation, Methodology, Project administration, Resources, Software, Validation, Visualization, Writing – original draft, Writing – review & editing. SP: Data curation, Formal analysis, Investigation, Project administration, Resources, Supervision, Validation, Visualization, Writing – original draft, Writing – review & editing.
